# Shell disease syndromes of decapod crustaceans

**DOI:** 10.1111/1462-2920.16344

**Published:** 2023-02-09

**Authors:** Andrew F. Rowley, Christopher J. Coates

**Affiliations:** ^1^ Department of Biosciences, Faculty of Science and Engineering Swansea University Swansea UK; ^2^ Department of Zoology, School of Natural Sciences Zoology, Ryan Institute School of Natural Sciences, University of Galway Galway Ireland

## Abstract

The term shell disease subsumes a number of debilitating conditions affecting the outer integument (the carapace) of decapod crustaceans, such as lobsters and crabs. Herein, we seek to find commonality in the aetiology and pathology of such conditions, and those cases that result in the progressive erosion of the cuticle through to the visceral tissues by a cocktail of microbial‐derived enzymes including lipases, proteases and chitinases. *Aquimarina* spp. are involved in shell disease in many different crustaceans across a wide geographical area, but the overall view is that the condition is polymicrobial in nature leading to dysbiosis within the microbial consortium of the damaged cuticle. The role of environment, decapod behaviour and physiology in triggering this disease is also reviewed. Finally, we provide a conceptual model for disease aetiology and suggest several avenues for future research that could improve our understanding of how such factors trigger, or exacerbate, this condition.

## INTRODUCTION

The outer integument of decapod crustaceans consists of acellular layers, with several containing the structural polysaccharide, chitin, chitoproteins toughened with calcite and an underlying epithelium (Dillaman et al., 2013). Chitin is the second most abundant natural polysaccharide after cellulose and it is also formed by insects and fungi. It is a homo‐polymeric carbohydrate consisting of repeating *N*‐acetyl‐β‐d‐glucosamine (GlcNAc) units that form the vital structural scaffold for the decapod exoskeleton. Chitin's accumulation in the marine environment is avoided by the action of abundant chitinolytic (chitinoclastic) microbes, notably bacteria (Keyhani & Roseman, 1999; Kumar et al., 2022). Its degradation by diverse chitinases yields carbon and nitrogen products, which represents large‐scale biochemical recycling of nutrients in marine food webs (Beier & Bertilsson, 2013; Souza et al., 2011). Numerous bacteria (e.g., aeromonads, vibrios) can sense and recognize chitin, adhere to the chitinaceous exoskeleton and hydrolyse the chitin into saccharides (Aunkham et al., 2018; Hunt et al., 2008; Zobell & Rittenberg, 1938). The enzymatic machinery needed to catabolize chitin and to re‐anabolise the products into biological materials is located extracellularly, in the bacterial cell envelope, periplasmic space and inner membrane, as well as the cytoplasm (reviewed by Keyhani & Roseman, 1999). Indeed, the chitin‐binding proteins and chitinases of pathogens such as *Vibrio cholerae* ensure their environmental survival (Frederiksen et al., 2013; Pruzzo et al., 2008). Both chitin and its deacetylated breakdown product, chitosan, are highly valued for use in the biomedical (Feng et al., 2021; Jayakumar et al., 2011) agricultural (El Hadrami et al., 2010), environmental (Tran et al., 2015) and food (Ngo et al., 2015) industries.

Because of the physicochemical composition of the crustacean carapace, it is highly resistant to desiccation and provides protection from predation, parasites/pathogens and poisoning (e.g., Dillaman et al., 2013; Moret & Moreau, 2012). Indeed, few microbes can penetrate the intact integument and those with such ability include certain oomycetes and fungi (e.g., Coates et al., 2022). Instead, viruses, some bacteria and protists gain entry to the internal tissues of their hosts via the alimentary canal or genital openings (Coates et al., 2022). The arthropod cuticle responds to the presence of invading pathogens by the activation of the prophenoloxidase cascade that results in the formation of antimicrobial factors and melanin (Cerenius & Söderhäll, 2004). This action may limit the invasive activity of pathogens like the oomycete, *Aphanomyces astaci*, which causes crayfish plague (Rowley et al., 2022). The cuticle of the American lobster (*Homarus americanus*) also contains small (<10 kDa) organic molecules (possibly antimicrobial peptides) that inhibit the growth of certain Gram‐positive and Gram‐negative bacteria (Brisbin et al., 2015). Pathogens and parasites that manage to gain entry into the haemocoel will usually elicit a strong host response involving the circulating haemocytes (immune cells of arthropods). A common reaction is to wall off parasites—a process termed encapsulation—in an attempt to limit their spread and colonization of the host. Some pathogens have mechanisms to circumvent, inhibit or evade this host response giving them access to other tissues (Coates et al., 2022; Rowley, 2016).

Varied reports of cuticle abnormalities of crustaceans—collectively called shell disease—date back to the 1930s (Hess, 1937). Alternate names for such conditions include shell disease syndrome, ‘classical’ shell disease, black spot, rust spot disease, box burnt disease, bacterial shell disease, tail fan necrosis, impoundment shell disease and brown spot disease (Davies & Wootton, 2018; Dyrynda, 1998; Getchell, 1989; Rowley et al., 2022; Shields, 2013; Vogan et al., 2008). Not all these conditions share the same aetiology and Table 1 summarizes three distinct forms, which for the purpose of clarity, are termed Types 1–3. Type 1 is the most observed across a range of crustaceans and is characterized by the progressive erosion of the cuticle by the action of microbial enzymes including proteases, lipases and chitinases (Bell et al., 2012). Types 2 and 3 do not always coincide with cuticle erosion, thereby representing distinct forms of the disease (Table 1). For instance, Type 3 shell disease comes about as a result of altered pigmentation in the cuticle while Type 2 reflects the host response to the presence of invading fungi and oomycetes. Due to the absence of carapace embrittlement, Types 2 and 3 are not considered ‘true’ shell diseases and will not be discussed further.

**TABLE 1:** Three main forms of shell disease affecting crustaceans.

Types	Type 1: erosion of cuticle	Type 2: Integumentary response to pathogen invasion usually without cuticle erosion	Type 3: changes in pigment but with no erosion of cuticle
Common names	Shell disease (including winter impoundment disease, endemic and epizootic shell disease	Burn spot disease	Rust spot disease
Main features	Progressive erosion of cuticle	Melanization reactions observed on the surface of the cuticle caused by microbial penetration of the cuticle, little or no cuticular erosion	Altered pigmentation but with no cuticular erosion
Microbial involvement	+ (changes in bacterial community structure)	+ (penetration of cuticle by fungi and/or oomycetes)	? (unknown aetiology—metal pollutant‐induced?)
Crustaceans affected	Wide range including lobsters, crabs	Crustaceans susceptible to fungal/oomycete penetration of cuticle including shrimp and crayfish	Narrow range including mud crabs (*Scylla serrata*) in Australia
Examples	Endemic shell disease in edible crabs (*Cancer pagurus*), winter impoundment shell disease and epizootic shell disease in American lobsters (*Homarus americanus*). See also Table 2	Penetration of shrimp and crayfish cuticles by *Fusarium* spp.	‘Rust spots’ in mud crabs
Key references	Vogan et al. (2008); Castro et al. (2012); Shields et al. (2012)	Makkonen et al. (2013); Rowley et al. (2022); Yao et al. (2022)	Andersen et al. (2000); Dennis et al. (2016)

In the years since the last in‐depth reviews of shell disease (Castro et al., 2012; Getchell, 1989; Gomez‐Chiarri & Cobb, 2012; Vogan et al., 2008), there have been major advances in our understanding of the erosive forms described as Type 1 (Table 1) that have not been collectively reviewed. For example, the emergence of epizootic shell disease (ESD) in American lobsters in the late 1990s continues to accompany declines in lobster stocks in the north‐eastern region of the USA. This initiated comprehensive research programmes, including the New England Lobster Research Initiative: Lobster Shell Disease, on the causes and consequences of this condition (reviewed by Davies & Wootton, 2018; Castro et al., 2012 and Gomez‐Chiarri & Cobb, 2012). More recently, this condition has spread northwards into the Gulf of Maine (Reardon et al., 2018), but to the best of our knowledge, there are no reports of its presence across the border into Canadian waters. Research efforts and opinions have moved forward since the reviews of Castro et al. (2012) and Gomez‐Chiarri and Cobb (2012), in particular, our understanding of microbial community ecology in shell disease lesions revealed by the development of multi‐omic technologies. The aim of this review is therefore to synthesize contemporary evidence of Type 1 shell disease with reference to the microbial ecology and the aetiology of this condition. Furthermore, rather than concentrate on one form of this condition in a single host species, we seek to provide a unifying overview.

## TYPE 1 SHELL DISEASE DISPLAYS VARIABLE SEVERITY, PREVALENCE AND PATHOLOGY

**FIGURE 1:**
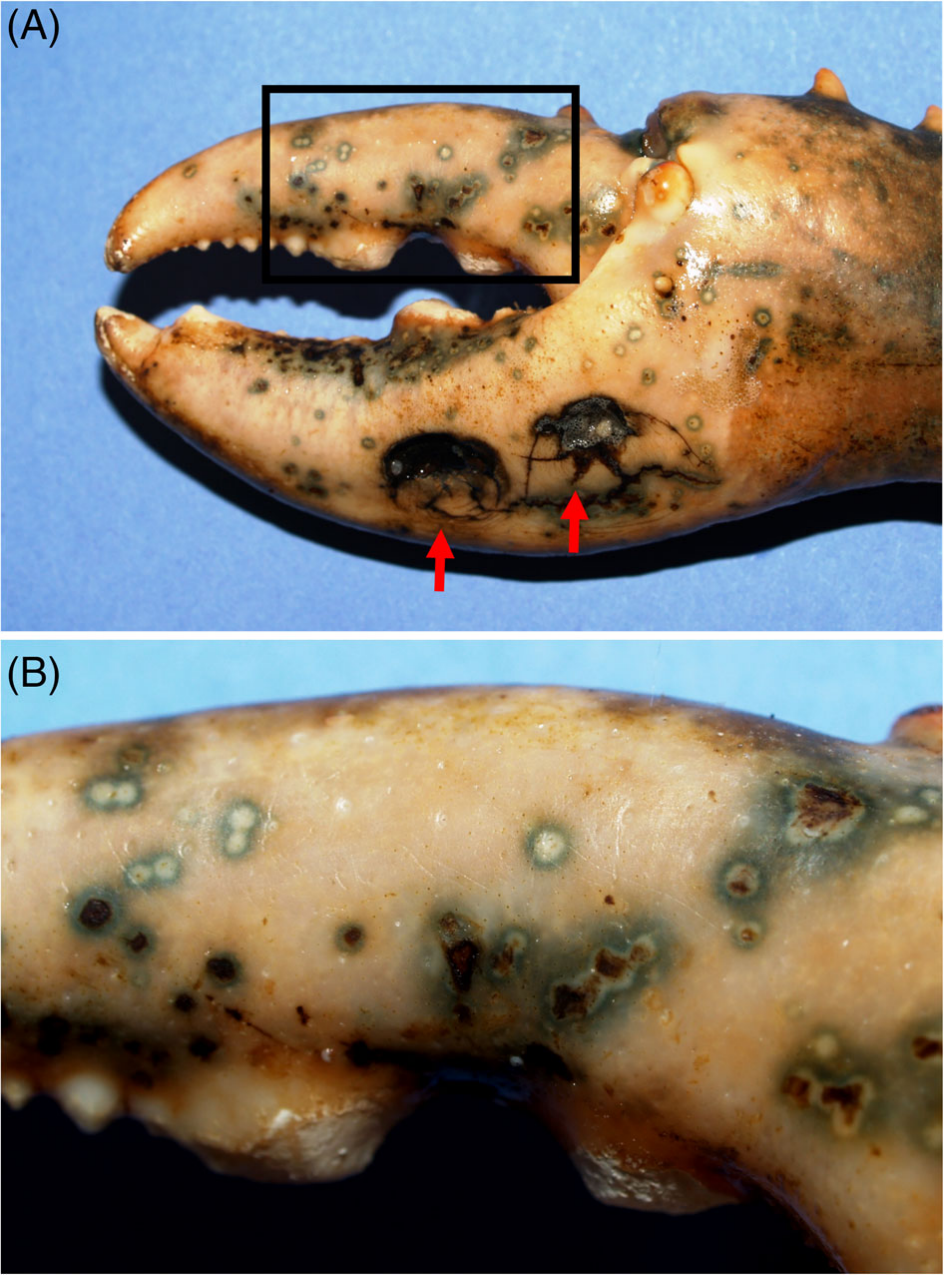
Shell disease lesions seen on claws of European lobsters, *Homarus gammarus* (A). (B) Magnified area in boxed region of (A) showing the nature of pin‐point lesions some with or without melanization. Micrographs courtesy of Drs. E. Wootton and C.L. Vogan.

**FIGURE 2:**
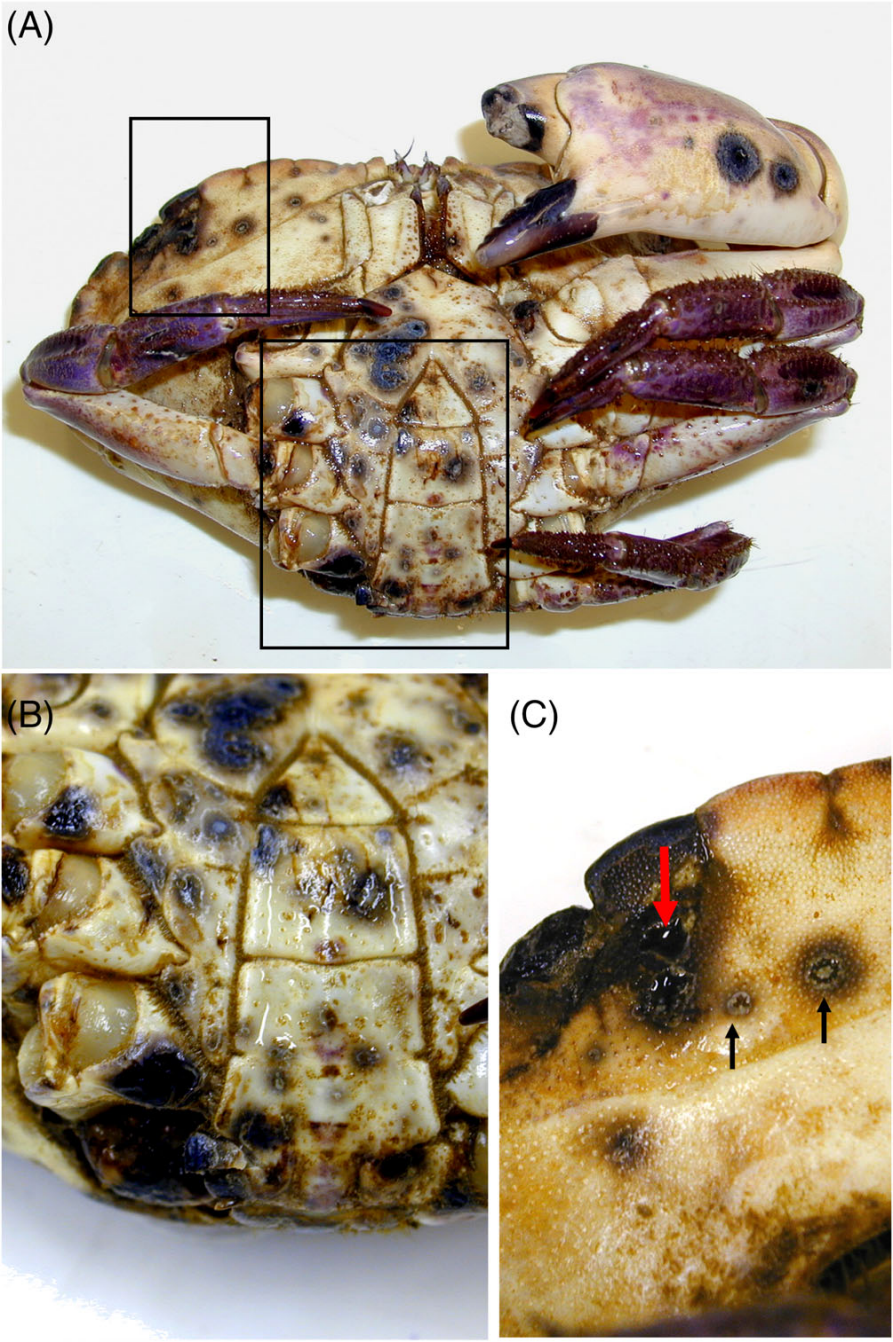
(A–C) Shell disease on ventral carapace of an adult edible crab, *Cancer pagurus*. The boxed areas in (A) show the regions magnified in Figures 2 B and C. Note superficial small (black arrows) and deeper larger (red arrow) lesions. Micrographs courtesy of Dr. Adam Powell.

Over 15 species of crustaceans are affected by Type 1 shell disease, including crabs, lobsters and shrimp, but the prevalence and intensity of disease varies. For instance, the common form of shell disease termed enzootic (= endemic) or ‘classical’ shell disease in European lobsters (*Homarus gammarus*) is often of low severity with small, pitted lesions on the claws (Figure 1A, B) but sometimes is found at high prevalence in natural populations (Davies et al., 2014, 2015; Wootton et al., 2012). A similar condition observed in edible (brown) crabs (*Cancer pagurus*) exceeds 76% in juvenile males (South Wales, UK) with lesions covering 0.8% of the carapace (Vogan et al., 1999, 2002; Figure 2A‐C). At a second location further north at the Isle of Man, UK, adult edible crabs landed from commercial vessels also exhibited high levels of shell disease ca. 24% (King et al., 2014), demonstrating high prevalence in crab populations in different locations. Unlike the lesions seen in lobsters, those in edible crabs occur over the entire carapace as well as the appendages (Figure 2). Interestingly, the prevalence and severity of this disease in crustacean populations co‐inhabiting the intertidal zone can be markedly different. For example, populations of European shore crabs (*Carcinus maenas*) and velvet swimming crabs (*Necora puber*) surveyed in Swansea Bay, UK, between 2017 and 2018 rarely exhibit shell disease (<1%), and if present, it is low intensity with only one or two discreet lesions per infected animal, while co‐located juvenile edible crabs were more heavily infected (unpublished observations associated with Davies et al., 2019). It remains unclear why some species are more resistant or susceptible to this condition.

Great emphasis has been placed on various forms of shell disease in the American lobster because unlike the endemic forms of disease already described that have limited economic effects, the emergence of highly destructive ESD in the western Long Island Sound, USA in the late 1990s coincides with population declines (Castro et al., 2012; Groner et al., 2018; Shields, 2013). Indeed, studies point to ESD as a driver of such changes as ovigerous (egg carrying) females that have long intermoult periods can show a high prevalence of this condition (>70%) resulting in their mortality (Hoenig et al., 2017; Shields, 2013). Furthermore, ESD may have sublethal effects on the reproductive fecundity of infected lobsters. To explore this, Gutzler et al. (2022) carried out a series of laboratory‐based studies on mating success of ESD infected and healthy lobsters. Female lobsters with active shell disease received a significantly lower volume of ejaculate from males compared to their healthy counterparts. However, in mating choice trails between healthy versus ESD‐affected females, males showed no preference suggesting that they could not discriminate diseased individuals by olfactory cues. Ostensibly, ESD has limited effect on the reproductive success of affected lobsters (Gutzler et al., 2022), but longer‐term consequences, such as larval survival, were not determined.

There are several distinct forms of Type 1 (classical) shell disease in American lobsters that are identifiable by external appearance and pathology. Table 2 summarizes the main differences in the potential aetiology and pathology of these conditions. As well as ESD, American lobsters suffer from at least four other forms, including enzootic, black spot, diet‐induced and winter impoundment shell diseases (Table 2; Quinn, Cawthorn, et al., 2013; Smolowitz et al., 2014). The gross appearance of the lesions differ but lobsters with high‐severity infections of these different forms may all look similar due to lesion coalescence and necrosis (Smolowitz et al., 2014), thereby resulting in potential confusion. Whether all these forms are truly distinct in terms of the aetiology of disease is unclear.

**TABLE 2:** Characteristics of potential different forms of shell disease in American lobsters (based on Chistoserdov et al., 2012; Shields et al., 2012; Smolowitz et al., 1992, 2005, 2014).

Features	Epizootic shell disease (ESD)	Impoundment shell disease (and endemic/enzootic shell disease?)	Black spot/trauma‐induced shell disease
Prevalence	High (>70% in ovigerous females in some areas)	Low (<1%)	Low
Gross appearance & pathology	Dorsal, deep, irregular lesions on the carapace, symmetric, melanised lesions with pillars of non‐eroded chitin. Haemocyte infiltration underlying cuticle	Variable appearance including rounded, melanised lesions, bilaterally symmetrical emerging from openings in cuticle. May exhibit extensive degradation of cuticle in severe cases giving a ‘scooped out’ appearance	*Trauma*: Heavily melanised irregular lesions on posterior abdominal segments
Potential environmental trigger(s)	Increase in sea temperature, anthropogenic chemicals (e.g. alkylphenols) resulting in moulting phenology and immune suppression	*Impoundment*: overcrowding, poor water quality, poor diet resulting in stress *Enzootic*:?	Lobsters found in degraded environments affected by sewage disposal.
Microbial composition of lesions	Bacterial dysbiosis including increase in numbers of *Aquimarina* ‘*homari’*, *Pseudoalteromonas gracilis* and *Thalassobius* sp. and other genera	Bacterial dysbiosis: *Impoundment*: *A*. *‘homaria’*, *A*. *macrocephali*, *Thalassobius* sp., *Tenacibaculum* spp., Candidatus *Homarophilus dermatus* *Enzootic*: *Tenacibaculum* spp., *Thalassobius* sp. *Thalassobacter* sp. *Aquimarina ‘homari’*. and several unidentified Gammaproteobacteria	*Trauma*: *Vibrio cyclitrophicus*, *Vibrio* sp., *Pseudoalteromonas tetraodonis*, *P*. *nigrifaciens*, *Aquimarina muelleri* and other *Flavobacteriaceae*
Key references	Smolowitz et al. (2005); Chistoserdov et al. (2005), Chistoserdov et al., 2012); Meres et al. (2012); Shields et al. (2012)	Bullis (1989); Smolowitz et al. (1992); Chistoserdov et al. (2012); Quinn Cawthorn, et al. (2013)	Quinn Cawthorn, et al. (2013)

There are no reports of ESD in European lobsters, *H*. *gammarus* in their native range despite increasing numbers of non‐native *H*. *americanus* found in Europe due to the importation and accidental release of live lobsters in certain areas (Øresland et al., 2017; Stebbing et al., 2012). To assess the risk of this practice to European lobsters, Whitten et al. (2014) co‐incubated American and European lobsters in an aquarium where ESD was present. They found no clear evidence that European lobsters were susceptible to this condition and other morphological studies on the carapace architecture of these two species found differences that may explain the apparent resistance to ESD. Specifically, a series of electron micrographs revealed the main layers (epi‐, exo‐ and endo‐cuticle) of lobster carapace are almost two‐fold thicker for *H*. *gammarus* than *H*. *americanus* (Davies et al., 2014). There are reports of non‐native American lobsters exhibiting symptoms like those of ESD housed in a public aquarium in Europe (van der Meeren, 2008) showing the apparent presence of this condition in European waters (these animals were caught locally). The susceptibility of hybrids of *H*. *americanus x gammarus* (Ellis et al., 2020) to ESD is also undetermined.

## TYPE 1 SHELL DISEASE IS A POLYMICROBIAL CONDITION LINKED TO BACTERIAL DYSBIOSIS AND THE PRESENCE OF *AQUIMARINA* SPP.

Initial studies designed to elucidate the aetiology of shell disease were carried out using traditional microbiological approaches including growing microbes isolated from lesions using culture media, such as chitin‐containing agars. Various teams concluded that a range of Gram‐negative bacteria (e.g., *Vibrio* spp.) were principally responsible for such conditions (Malloy, 1978; Mancuso et al., 2010; Noga et al., 1994). Most of these ‘early’ studies attempted to identify isolates using phenotypic and biochemical approaches like those in commercially available API identification kits—principally designed to identify pathogens of humans and common environmental isolates (e.g., Vogan et al., 2002). Unfortunately, these approaches have clear limitations as not all members of the microbial community grow on generalist agars and identification using these methods is tentative. Vogan et al. (2002) isolated and characterized chitinolytic bacteria found in shell disease lesions in *C*. *pagurus* and many were considered vibrio‐like in terms of their biochemical activities. Detailed studies on some of these isolates found that they were pathogenic upon intrahaemocoelic injection and used putative virulence factors (toxic extracellular products; Vogan et al., 2002; Costa‐Ramos & Rowley, 2004). However, no crabs from natural populations have been found infected by any of the bacterial isolates.

Chistoserdov et al. (2005) also used a culture‐dependent approach to examine lesions from lobsters with ESD. Instead of relying on biochemical approaches to characterize isolates, they employed 16 S rRNA sequencing to identify ‘representative’ isolates found in the lesions. Although finding vibrios and pseudomonads as reported in other studies of shell disease, they revealed new species including members of the family *Flavobacteriaceae*, a novel *Aquimarina* species (originally named *A*. *‘homari’*) and *Pseudoalteromonas gracilis* together with other less frequently observed isolates like *Vibrio lentus*, *Pseudoalteromonas tunicata*, *Shewanella fidelia* (*fidelis*; Ivanova et al., 2003; Thorell et al., 2019), and other vibrios. Levels of bacteria in the lesions were several fold higher than in healthy carapace scrapings. The potential pitfall of this initial key study was that it relied on the ability of bacteria to grow on various media that may not emulate the dynamic bacterial community in lesions. Later culture‐independent approaches verified those findings, however, with the addition of another novel member of the family *Rhodobacteraceae*, related to *Thalassobius* spp. (Chistoserdov et al., 2009; Chistoserdov et al., 2012).

*Aquimarina* sp. *‘homaria’* (later reclassified as *A*. *macrocephali* subsp. *homaria*; Quinn et al., 2017 and *Aquimarina* sp. strain 132–134; Ranson et al., 2018), *P*. *gracilis* ISA7.3 and *Thalassobius* sp.131.1 (Ranson et al., 2019) are associated with lesions from ESD affected lobsters (Chistoserdov et al., 2005). It is noteworthy that *Aquimarina* spp. (including *A*. sp. *‘homari’*) is found on healthy cuticle and absent from some ESD lesions (Meres, 2016; Meres et al., 2012; Whitten et al., 2014). Aquarium‐based studies demonstrated that abraded healthy lobsters, *H*. *americanus* when placed in direct contact with *A*. *‘homari’* and *Thalassobius*, developed lesions on the cuticle (Quinn, Metzler, Smolowitz, et al., 2012), yet abrasion is required for the initiation of lesions. Abrasion likely removes the epicuticle—an outer waxy layer devoid of chitin—although, it is unclear how it becomes compromised in natural infections. In the same study, *P*. *gracilis* did not induce lesion formation, indicating it is a secondary colonizer during lesion formation (Quinn, Metzler, Smolowitz, et al., 2012). Quinn et al. (2017) developed a specific PCR method to study the distribution of *A*. *‘homari’*. Using this approach, they confirmed *A*. *‘homari’* resided in lesions at much higher levels than on healthy carapace at least in *H*. *americanus* with ESD. Although it was found on other crustaceans, such as spider crabs (*Libinia emarginata*), shore crabs (*C*. *maenas*) and Jonah crabs (*Cancer borealis*), levels of *A*. *‘homari’* in lesions did not significantly differ from healthy carapaces.

*Aquimarina* is a little‐studied genus of bacteria belonging to the family *Flavobacteriaceae* (Nedashkovskaya et al., 2006, 2015; Ooi et al., 2020). This is a group of Gram‐negative, rod shaped, strictly aerobic halophiles and many of the species are capable of chitin breakdown. Over 15 known species of the marine genus have been described, including *A*. *hainanensis*, which is pathogenic towards mud crab (*Scylla serrata*) and shrimp (*Penaeus vannamei*) larvae in hatcheries (Midorikawa et al., 2020; Zheng et al., 2016). Other species of *Aquimarina* are associated with diverse taxa including *A*. *intermedia* from sea urchins (*Strongylocentrotus intermedius*; Nedashkovskaya et al., 2006), *A*. *mytili* from mussels (*Mytilus coruscus*; Park et al., 2012), *A*. *spongiae* from sponges (*Halichondria oshoro*; Yoon et al., 2011) and *Aquimarina* spp. from red alga (*Delisea pulchra*; Hudson et al., 2019). Further species are reported in marine sediments (e.g., Lee et al., 2017), sea water (e.g., Choi et al., 2018; Wang et al., 2018; Yu et al., 2013; Zhang et al., 2014), and associated with plastic waste (Vidal‐Verdu et al., 2022). Three recent publications described an association between *Aquimarina* spp. and shell disease in tail fan necrosis in spiny lobsters (*Jasus edwardsii*; Zha et al., 2019), in shell disease in edible crabs, *C*. *pagurus*, and in white leg disease in larvae of spiny lobsters (*Panulirus ornatus*), eastern rock lobsters (*Sagmariasus verreauxi*) and slipper lobsters (*Thenus australiensis*; Ooi et al., 2020). Thus, this bacterium co‐occurs with various forms of shell disease (i.e., enzootic/endemic‐like and ESD) in diverse crustacean species across geographically distinct regions.

Researchers have attempted to characterize the bacterial lesion communities in different forms of shell disease in American lobsters (see Table 2) to elucidate if these are unlike ESD (Quinn, Cawthorn, et al., 2013). Using a series of oligonucleotide primers and denaturing gradient gel electrophoresis (DGGE) separation of amplicons, they observed bacterial communities of lesions from trauma and enzootic shell disease differed from each other and also from ESD. The main bacterial forms in trauma shell disease found in seven out of eight samples were vibrios (*V*. *cyclithotrophicus*, *Vibrio* sp.) and pseudoalteromonads (*P*. *nigrifaciens*, *P*. *tetraodonis*), although *Aquimarina* spp. were present in fewer samples (Quinn, Cawthorn, et al., 2013; Table 2). Lesion samples from lobsters with enzootic shell disease principally contained two species of *Tenacibaculum* (*T*. *lutimarus*, *Tenacibaculum* sp.) that also belong to the family *Flavobacteriaceae*. Other *Tenacibaculum* are pathogens of many marine fish causing tenacibaculosis, with its characteristic ulceration around the mouth (Avendaño‐Herrera et al., 2006; Nowlan et al., 2020). Quinn, Cawthorn, et al. (2013) did not find *Aquimarina* spp. in any of the lesion samples from lobsters in Canadian waters with enzootic shell disease (*n* = 13), yet Chistoserdov et al. (2012) found them in two lobsters with the syndrome off Rhode Island, USA.

Clearly, *Aquimarina* spp. are not the only primary pathogens of ESD and that this, and other forms of ‘Type 1’ shell disease, are not examples of the long‐held fundamental concept of ‘one microbe, one disease’—hence Koch's postulates cannot be satisfied. Within invertebrate pathology, there is an increasing focus on the concept of the ‘pathobiome’ (Bass et al., 2019), and several disease conditions ranging from those in corals to more advanced aquatic invertebrates, appear to be multi‐factorial in nature involving more than one microbe/parasite often with an environmental driver (Bourne et al., 2022; Petton et al., 2021; Sweet & Bulling, 2017). Shell disease is currently considered a dysbiotic condition resulting from changes in epibiotic (microbial) populations (Bergen et al., 2022; Chistoserdov et al., 2012; Feinman et al., 2017; Meres, 2016; Meres et al., 2012). As well as ESD already described, tail fan necrosis of spiny lobsters is characterized by lesions with a consortium of microbes, including *Flavobacterium*, *Streptomyces*, *Neptunomonas*, other *Flavobacteriaceae* and *Thiohalhabdales*, as well as *Aquimarina* (Zha et al., 2019). These lesion communities show significant reductions in richness, diversity and evenness when compared to healthy cuticle, and reinforce the concept that shell disease is an example of a dysbiotic condition. Recently, Bergen et al. (2022) showed that shell disease lesions in *C*. *pagurus* caught in the Baltic Sea have reduced microbial community composition with an increase in *Aquimarina* spp. Overall, shell disease is one of an expanding group of conditions displaying the polymicrobial nature of invertebrate pathologies (Bass et al., 2019; Munkongwongsiri et al., 2022; Petton et al., 2021; Sharma & Ravindran, 2020).

An exception to the ‘polymicrobial view’ is an unusual form of shell disease, white leg disease, caused by a single species of microbe independent of other pathogens. This cuticular disease affects larval stages of several species of lobsters in cultivation. Both traditional culture‐dependent and culture‐independent analyses showed the presence of a dominant microbe, *Aquimarina* sp. (TRL1), which shared 100% sequence homology with *A*. *hainanensis* (Ooi et al., 2020). Exposure of lobster larval stages to the isolate for 30 min caused infections with similar pathologic signs to the disease. Notably, for this infection to be attained, the lobsters needed initial cuticular damage, which prompted Ooi et al. (2020) to conclude that injury to lobster pereiopods predisposed them to this condition.

## THE CASE FOR CHITINASES AS VIRULENCE FACTORS IN SHELL DISEASE

**FIGURE 3:**
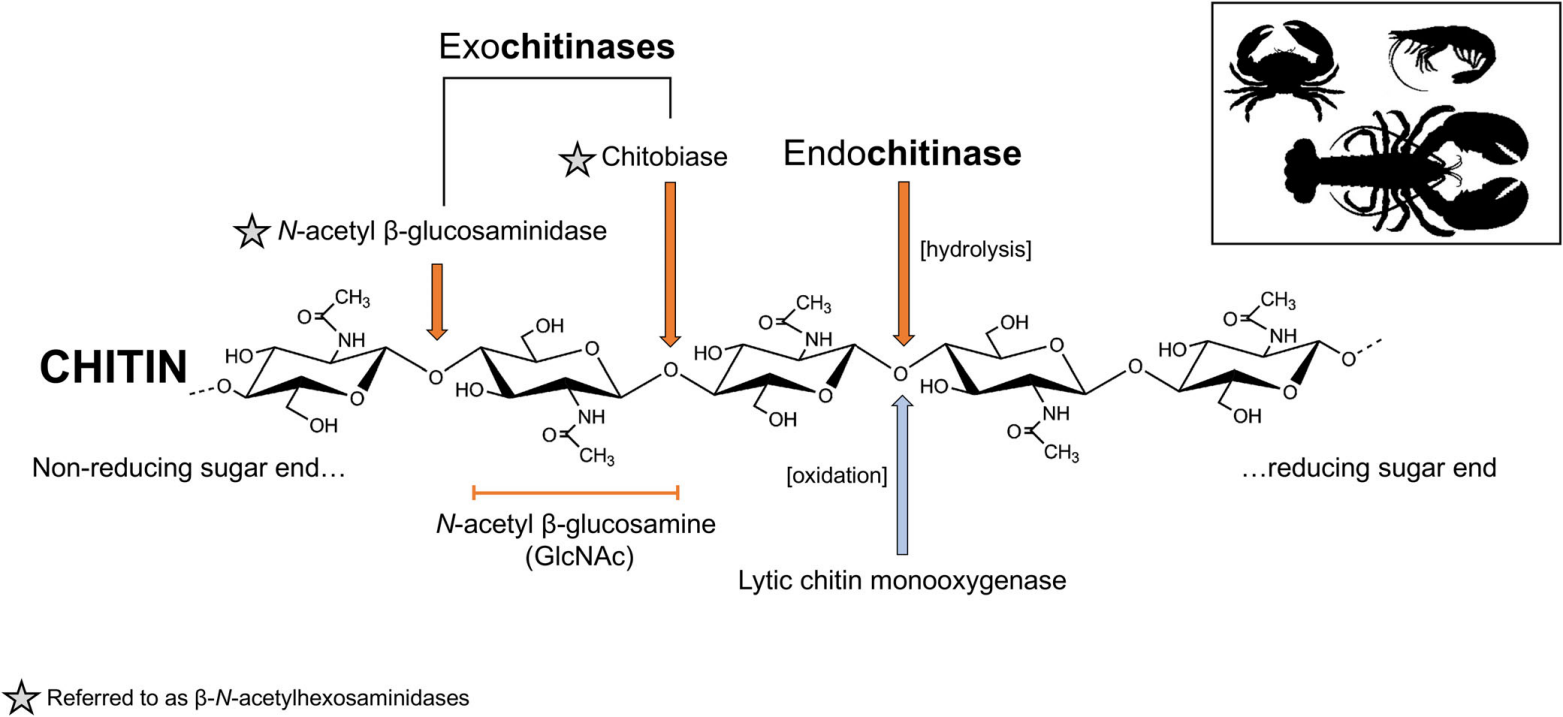
Enzymatic breakdown of chitin associated with shell disease. The orange and blue arrows indicate enzyme‐mediated hydrolysis and oxidation as the catalytic mechanisms, respectively. Endochitinases can act on random glycosidic bonds along the chitin, generating free ends onto which exochitinases can process. Chitin degradation is enhanced by the presence of lytic chitin monooxygenase.

The oceans are replete with chitinovorous microbes (Swiontek‐Brzezinska et al., 2007; Zobell & Rittenberg, 1938), which can be pathogens or detritivores, hence targeting living crustaceans as opposed to cadavers, discarded moults, or autotomized limbs. We know relatively little about the virulence factors of bacteria isolated recurrently from carapace lesions, perhaps due to the lack of defined primary causative agents of shell disease. As introduced earlier, microbial‐derived endo‐chitinases (i.e., 1,4‐β‐poly‐*N*‐acetylglucosaminidase; EC 3.2.1.14) and exo‐chitinases (i.e., β‐*N*‐acetylhexosaminidases; EC 3.2.1.52) hydrolyse the glycosidic bonds that make‐up chitin microfibrils and chitodextrins (Figure 3). Bacterial endo‐ and exo‐chitinases are grouped into the glycoside hydrolase families 18 (class V) and 20, respectively (Chen et al., 2020). It is worth noting that chitobiase (or chitobiosidase; formerly EC 3.2.1.29) and *N*‐acetyl β‐glucosaminidase (formerly EC 3.2.1.30) are now grouped together as β‐*N*‐acetylhexosaminidases.

First, the bacterial endochitinases randomly cleave chitin at internal sites to yield soluble, low molecular weight derivatives, that is, di‐acetylchitobiose, chitotriose, and chitotetraose, with the former being the most common. These chitooligosaccharides possess non‐reducing (free) ends where exochitinases can bind and further breakdown into individual GlcNAc units. Another enzyme, lytic chitin monooxygenase (or chitin oxidohydrolase; EC 1.14.99.53), disrupts glycosidic bonds through oxidative degradation reactions, which assist in depolymerising the recalcitrant chitin, and makes it more accessible for endochitinases. Chitin fibrils are also targeted by de‐acetylase (EC 3.5.1.41), which forms chitosan and acetate (C_2_H_3_O_2_^−^). Chitosan is cleaved via endohydrolysis into glucosamine units (GlcN) by a diverse group of chitosanases (EC 3.2.1.132). Theoretically, the concurrent release of lipases to degrade the epicuticular waxes, and proteases to degrade structural proteins and potential anti‐infective factors (antimicrobial peptides, antioxidant enzymes) likely occurs in a similar manner to the cocktail of enzymes released by entomopathogenic fungi making their way across the insect integument (reviewed by Butt et al., 2016). The use of diverse chitinases by insect killing fungi and bacteria as virulence factors is well established in terrestrial systems (e.g., Fan et al., 2007; Huang et al., 2016; Sampson & Gooday, 1998).

Genome sequencing of *Aquimarina* sp. strain I32.4—an attendant microbe of lobster ESD—revealed 16 putative chitinase genes (Ranson et al., 2018), which positions *Aquimarina* spp. as candidate primary colonizers (or initiators) of shell disease. Conversely, the drafted genome of *Thalassobius* sp. I31.1—another purported marine pathogen—contained no chitin catabolic genes (Ranson et al., 2019). We can speculate that *Thalassobius* spp. are examples of secondary (or opportunistic) colonizers of shell disease. To the best of our knowledge, a temporal study of chitinase‐associated gene/protein expression, or microbial metatranscriptomics, at the onset and progression of shell disease lesions has not been published. Such a study would advance our understanding of the key players and factors driving this disease or rule out taxa that are merely associated with healthy versus diseased carapace. Beyond dissolving chitin, chitinases display non‐enzymatic functions, such as quorum sensing (e.g., Defoirdt et al., 2010). Perhaps it is this regulation of activity that attracts different waves of would‐be colonizers to the shell disease lesions.

## ENVIRONMENTAL, PHYSIOLOGICAL AND BEHAVIOURAL TRIGGERS OF SHELL DISEASE

Snieszko (1973) and others were first to highlight the importance of environment, together with host and pathogen/parasite biology, in what has become a classic triad of a Venn diagram, where the overlap represents disease. It is now obvious that many aquatic diseases are either caused, or exacerbated by, environmental factors (e.g., Coates & Söderhäll, 2021). Therefore, it is hardly surprising that shell disease as a condition of unknown aetiology, may be another example of a disease where environmental factors play a major role. The following section summarizes the environmental, physiological and behavioural triggers of shell disease. We provide a mechanistic explanation of how environmental changes can result in this condition both in different hosts and distinct geographic regions.

### Abrasion and fighting behaviour as triggers of shell disease

Aquarium‐based studies have shown that abrasion is a requirement for the experimental initiation of some forms of shell disease (e.g., Ooi et al., 2020; Whitten et al., 2014). The outer layer of the crustacean carapace is the epicuticle, a thin layer containing lipoproteins and calcite (Roer & Dillaman, 1984), and is likely responsible for controlling the formation of microbial biofilms on the surface of the crustacean. Breaching of this layer exposes chitin in the underlying exo‐ and endo‐cuticle leaving animals vulnerable to the chitinolytic activities of marine microbes (Rowley, 2022). Abrasion and fighting injuries are probably key determinants of shell disease in crustaceans in the wild and under culture conditions.

Several forms of shell disease are triggered by abrasion and/or fighting behaviours, for example, tail fan necrosis in spiny lobsters (*J*. *edwardsii*) and shell disease in on‐shore juvenile edible crabs (*C*. *pagurus*) and lobsters (*H*. *gammarus*, *H*. *americanus*). Tail fan necrosis is a bacterial condition of *J*. *edwardsii* characterized by a blackening and degeneration of the uropods and telsons. This condition affects both wild and captive lobsters, with capture handling practices promoting chance injuries along the delicate tail fan assemblage leading to this disease state (Bryars & Geddes, 2005; Freeman & MacDiarmid, 2009; Zha et al., 2018). Lobsters in marine reserves, where capture fishing is not allowed, show a reduced prevalence of tail fan necrosis (Freeman & MacDiarmid, 2009), presumably due to lower chances of mechanical damage.

Clearly, crustaceans grown in captivity require additional controls, such as reducing stocking density (and hence reducing fighting) through to individual holding as is the case of European lobsters, for both re‐stocking purposes and ranching (Hinchcliffe et al., 2022) to reduce the risk of lesion formation and progression.

**FIGURE 4:**
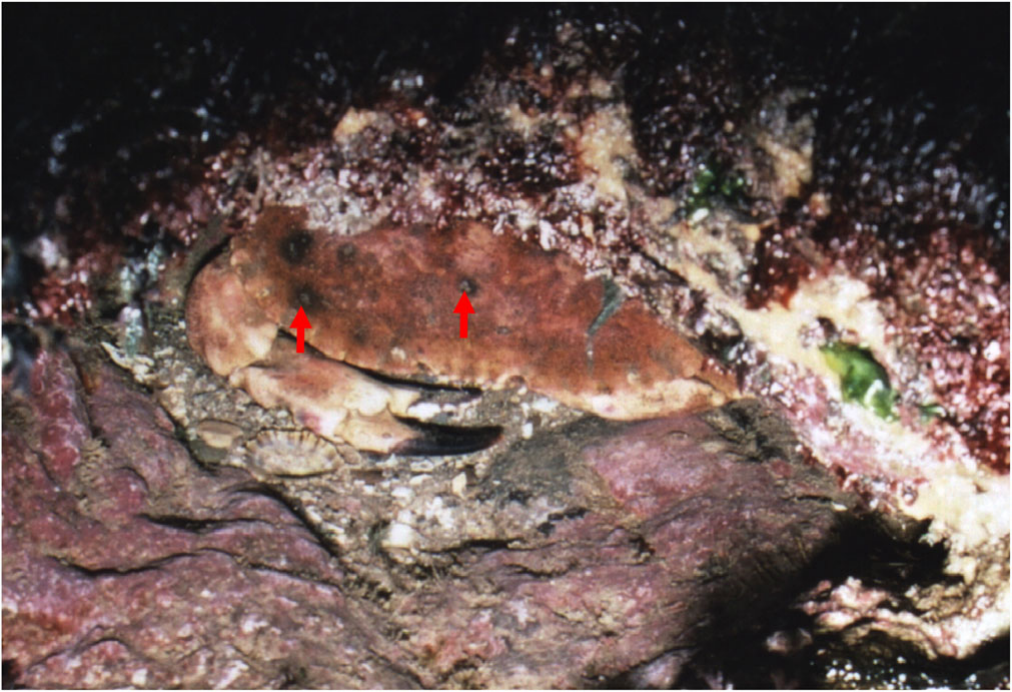
Back burrowed edible crab, *Cancer pagurus* photographed at Langland Bay, Gower, U.K. Note shell disease lesions visible on dorsal surface of the crab (unlabelled arrows). Micrograph courtesy of Dr. C. L. Vogan.

Vogan et al. (1999) examined the prevalence and intensity of shell disease in juvenile edible crabs, *C*. *pagurus*. These live in rock pools in the intertidal zone that are exposed at low tide. They back burrow into crevices as a defence mechanism to protect from predation (Figure 4) and this behaviour is probably responsible for the characteristic pattern of lesion development in the hind regions of these animals (Vogan et al., 1999, 2002). A correlation exists between shell disease prevalence and intensity, and the levels of chitinase‐producing bacteria in the sediment where these crabs live (Vogan et al., 2002)—reflecting the importance of abrasion with sediment in initiating this disease.

Finally, both American and European lobsters are subject to low levels of endemic (enzootic) shell disease in populations across the northern hemisphere. Both animals are largely solitary, living on the benthos or in crevices and are strongly territorial using their impressive pincer and crusher claws as weapons. Unsurprisingly, most of the discrete shell disease lesions found in such lobsters appear on the claws and many of these are damaged due to fighting injuries and general trauma resulting in limited erosion of the carapace (Figure 1). This form of the disease is superficial and unlikely to have any long‐term consequences to its host.

### Anthropogenic contaminants and shell disease

Several environmental contaminants have been proposed as cofactors in shell diseases, including ESD. As well as heavy metals (Weinstein et al., 1992) and sewage (Young & Pearce, 1975), much attention has focussed on alkylphenols (Jacobs et al., 2012; Laufer et al., 2013; Laufer, Baclaski, et al., 2012; Laufer, Chen, et al., 2012). These latter chemicals are incorporated in the manufacture and breakdown of plastics and other domestic products and have been detected in sewage outfalls and the aquatic environment where they can act as endocrine disruptors in some vertebrates (Annamalai & Namasivayam, 2015; Laufer et al., 2013). Alkylphenols have been discovered in the tissues of American lobsters in the waters off the USA where ESD is prevalent. For instance, the haemolymph (blood) of lobsters contained varying levels of three different alkylphenols but these were neither correlated with their size or ESD severity (Jacobs et al., 2012). As argued by the authors, the presence of alkylphenols in haemolymph is relatively transient (a few weeks) while shell disease is a long‐term condition, and hence this lack of correlation between disease severity and the levels of chemicals does not rule out the possibility of a link between earlier disease initiation and alkylphenol exposure.

Some alkylphenols are also incorporated into the cuticle of lobsters and can adversely affect sclerotization, which is responsible for hardening the new cuticle post‐moult (Laufer, Chen, et al., 2012). Injection of pre‐moult lobsters with an alkylphenol (2,4‐bis‐[dimethylbenzyl] phenol) caused a significant delay in the hardening of the new cuticle potentially leaving these animals vulnerable to microbial attack. Although a link between alkylphenols and the appearance of ESD is unproven, they may have a part to play in leaving lobsters vulnerable to a range of diseases as well as ESD that are problematic in the Long Island Sound and surrounding areas (Shields, 2013).

Other environmental contaminants, namely heavy metals, are contributory factors to shell disease. Higher levels of metals including aluminium and chromium have been  reported in blue crabs (*Callinectes sapidus*) with shell disease in the Pamlico Albemarle estuaries in the USA (Weinstein et al., 1992). A systematic study found no evidence that metals such as cadmium or mercury impacted lobsters with ESD (LeBlanc & Prince, 2012). Similarly, sewage pollution has also been linked to shell disease but generally without any direct evidence of cause and effect (Sawyer, 1991). The studies of shell disease in edible crabs (*C*. *pagurus*) in Langland Bay (Gower, UK) by Vogan and colleagues (Vogan et al., 1999, 2001, 2002; Vogan & Rowley, 2002) were from intertidal zones affected by the input of raw sewage waste. While it was not thought that the coliforms in such sewage were directly contributing to shell disease, the accompanying large amount of organic matter could stimulate more microbial growth in sediments. However, Powell and Rowley (2005) returned to the same survey site in Langland Bay several years after the regular pumping of raw sewage was terminated but found no evidence for any reduction in either the prevalence or severity of this condition pre‐ and post‐sewage influx.

### Changes in sea water temperature and the emergence of ESD

**FIGURE 5:**
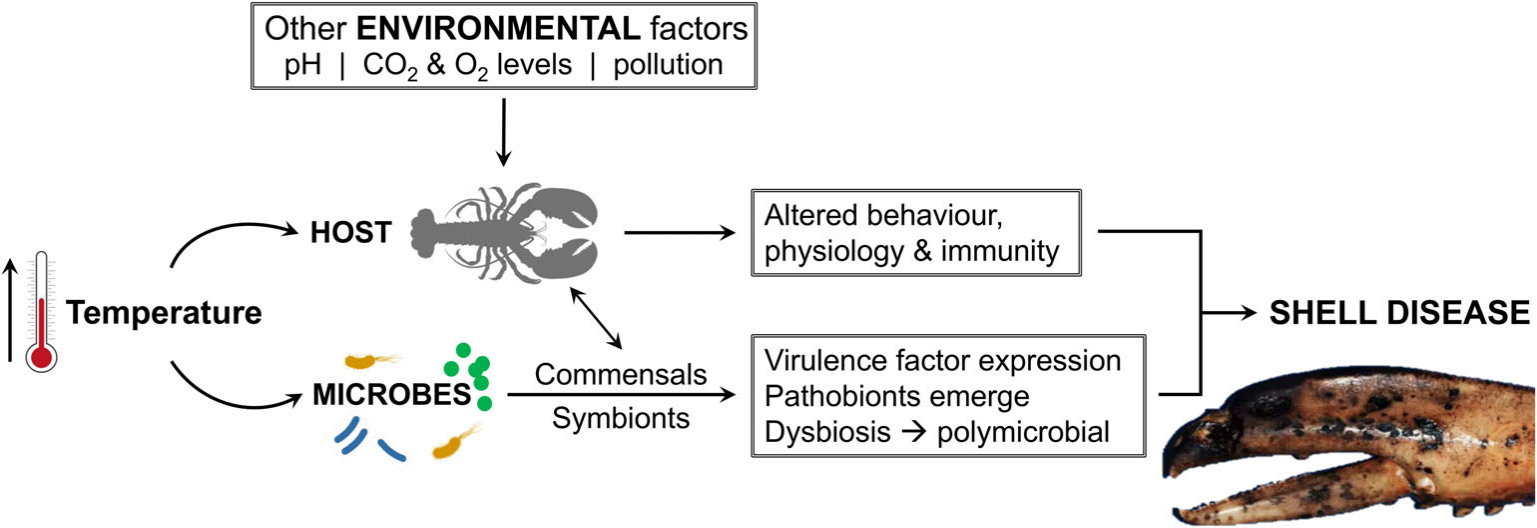
Conceptual model of how temperature (and other factors) may promote changes in both host and surface microbes leading to shell disease. Altered physiology of the host includes moulting frequency. Microbial consortia of shell disease lesions usually display lowered diversity and richness indices when compared to non‐diseased areas of carapace (i.e., they are dysbiotic).

Temperature changes caused by global warming are important drivers of disease in many aquatic organisms (see reviews by Harvell et al., 2002; Burge et al., 2014; Burge & Hershberger, 2020). Such changes can affect both the pathogen (e.g., multiplication rate, expression of virulence factors) and host (e.g., immune modulation and haemolymph acid–base balance). For instance, changes in the transcriptome of post‐larval American lobsters exposed to higher temperatures predicted to occur more frequently in future years, inhibit factors associated with cuticle formation and immune reactivity (Harrington et al., 2020). In most cases, higher temperatures coincide with heightened disease severity (Figure 5). Migration of pathogens/parasites and hosts caused by temperature perturbance can also bring about the potential for the spread of new (emerging) diseases.

The appearance of ESD in the 1990s in waters off southern Massachusetts and the Long Island Sound has been linked to the increased sea temperature of 1–2°C in these areas (Glenn & Pugh, 2006; Ishaq et al., 2022; Shields, 2013, 2019; Tanaka et al., 2017). At the southern margins of the range of American lobsters, these heightened temperatures will affect both host and microbial communities. In a key study, Shields (2019) modelled the thermal range of lobsters and a range of diseases including ESD. The optimum temperature for lobsters is approximately 12°C and above that they show progressive alterations in their metabolism, and based on data on ESD, temperatures above approximately 17°C favour its progression (Barris et al., 2018; Shields, 2019). So higher temperatures favour the progression of ESD to the detriment of the host.

Elevated temperature can affect several physiological and immunological processes in lobsters, and aquatic animals in general. The process of moulting is important in our understanding of shell disease as the timing and length of intermoult period (the time between moults) can influence the severity of this condition. The longer the intermoult period, the more time available for lesions to develop and in most cases moulting will result in the loss of the diseased carapace. Lobsters with ESD have higher moult frequencies and laboratory‐based studies show that apparently disease‐free animals held at 20°C have double the moulting rates than at 10°C (Tlusty & Metzler, 2012). The progression of ESD lesions is also faster at higher temperatures such as those experienced in areas where ESD abounds (Barris et al., 2018; Tlusty & Metzler, 2012). Moulting in lobsters is linked to season (i.e., it is a phenological event). The warmer winters in areas experiencing ESD result in an earlier spring moult in larger lobsters leaving a longer intermoult period before the next moult during which time the disease progresses (Groner et al., 2018).

The second effect of temperature on lobsters is a reduction in immune competence leading to increased susceptibility to disease. Populations of lobsters sampled from regions with and without ESD differ in terms of their immune capacity (Homerding et al., 2012). In detail, apparently healthy lobsters from Eastern Long Island Sound (an area with high levels of ESD) show reduced phagocytic activity by the circulating haemocytes and the killing mechanisms of these phagocytes were also reduced in comparison with those of cells taken from lobsters in areas with no or low ESD. Hence, these data imply that lobsters in those areas with high levels of ESD have reduced immune capacity but the cause of this may or may not be temperature linked.

As already discussed, there is mounting evidence that elevated sea surface temperatures in many areas are linked to ESD and other diseases (Shields, 2013), however, an explanation of the mechanism(s) that result in lobsters becoming susceptible to ESD is elusive. Perhaps a missing link to the puzzle about temperature as a trigger of ESD is its potential effects on those bacteria contributing to its aetiology. For example, is the growth of *A*. *‘homari’* increased in temperatures like those considered to favour disease? Similarly, is the synthesis of virulence factors of this bacterium and others important in ESD linked to temperature? In the simplistic model shown in Figure 5, those bacteria that may form part of a healthy carapace microbiome could have lower thermal ranges than bacteria in lesions. Their loss results in the dominance of *Aquimarina* spp. and other pathogenic bacteria, that overwhelm the commensal forms (symbionts) resulting in a lower microbial diversity and dysbiosis. Experiments focussed on bacteria from lesion communities may lead us closer to understanding the emergence of all forms of shell disease.

### Other environmental factors

The process of ocean acidification resulting from CO_2_ enrichment (i.e., H^+^ accumulation) has obvious effects on those animals with body coverings containing insoluble calcium salts. There is a plethora of published research on the physiological, biochemical and immunological effects of elevated CO_2_ on a range of marine invertebrates that are reviewed elsewhere (e.g., Bednaršek et al., 2021; Doney et al., 2009; Fabry et al., 2008; Whiteley, 2011). To date, the only investigation into elevated CO_2_ and crustacean shell disease comes from aquarium‐based studies by McLean et al. (2018). They reported that elevated CO_2_ levels of *p*CO_2_ 1000 and 2000 μatm affected the growth and moulting of lobsters, *H*. *americanus*. Furthermore, these conditions resulted in elevated levels of shell disease probably caused by increases in intermoult periods (McLean et al., 2018).

### Nutrition

As shell disease is mainly a condition of wild crustacean populations, it is difficult to ascribe its presence to poor or inadequate nutrition. One form of shell disease in American lobsters is impoundment disease when these animals are held for various periods before going to market. The lesions on lobsters with this disease can become extensive and may result in mortality (Table 2). During this time of short‐term holding (up to 6 months; Albalat et al., 2019), lobsters are not usually fed and so nutrition could be a contributory factor to their disease resistance during holding and the nutritional status of such animals entering impoundment may be of importance in calculating their risk of developing shell disease. Theriault et al. (2008) observed that animals with the lowest levels of protein in their haemolymph at the beginning of an experimental impoundment were the most likely to succumb to shell disease. Further risk factors like shell hardness and exposure to sludge were not as important as haemolymph protein levels in determining disease outcomes. Hence, the nutritional status of lobsters entering commercial impoundment is likely to be of importance in the outcome of this process and their general health and welfare (e.g., Albalat et al., 2019, 2022).

Poor diet is considered a contributory factor in ESD. Lobsters in heavily fished areas appear to have a higher proportion of finfish in diets resulting from the use of trash fish in bait. Bethoney et al. (2011) explored the concept that this restricted diet could be a factor in the emergence of ESD but concluded that this practice was not linked to this condition. Diet‐induced shell disease is an experimental condition in lobsters held in captivity receiving a diet based on fish alone (Quinn, Metzler, Tlusty, et al., 2012; Tlusty et al., 2008). Lobsters under such conditions develop lesions with microbial communities including *A*. *‘homaria’*, *Candidatus* Kopriimonas aquarianus, and *Kiloniella* sp. (Quinn, Metzler, Tlusty, et al., 2012). This form of shell disease is best regarded as artefactual and is unlikely to occur in wild populations of lobsters.

## CAN SHELL DISEASE LEAD TO SEPTICAEMIA AND HOST DEATH?

While shell disease is often superficial, and therefore, unlikely to penetrate through the cuticle to the underlying tissues, there is evidence that severe forms can result in bacterial colonization of the haemolymph (when the protective cuticle becomes compromised). Histopathological studies of various forms of Type 1 shell disease report damage in several internal tissues, including the digestive cells in the hepatopancreas (Comeau & Benhalima, 2009; Vogan et al., 2001), implying systemic changes caused by bacterial septicaemia. Vogan et al. (2001) showed that bacterial numbers in the haemolymph of edible crabs (*C*. *pagurus*) were correlated with the severity of shell disease lesions although the numbers of culturable bacteria.ml^−1^ were small (maximum 100 CFU ml^−1^ haemolymph) and the identity of the isolates was not investigated to ascertain if they were the same as the bacteria seen in the lesions. Although Shields et al. (2012) found higher numbers of vibrio‐like bacteria in the haemolymph of lobsters with ESD, the cause of this was most likely from fishing related and other injuries unrelated to shell disease. In a culture‐independent study of the haemolymph microbiome of lobsters with and without ESD the bacteria identified were found to be dissimilar to those in the lesion communities (Quinn, Somolowitz, & Chistoserdov, 2013). There was no gross correlation between the haemolymph microbiome and that of lesions suggesting a different source for the bacteria observed. Similarly, Jung et al. (2021) found that the haemolymph microbiome of newly dead lobsters (*H*. *americanus*) with ESD differed from that of lesions and was most similar to the bacterial communities found in the water in holding tanks. However, a complication from this study arises as the haemolymph samples were taken from animals 1–24 h post‐death that will result in *post‐mortem* changes associated with tissue autolysis. Finally, another form of shell disease termed white leg disease of larval lobsters, results in mortality in aquarium‐based experiments when these crustaceans are exposed to high numbers of putative pathogens (*Aquimarina* sp.) in the holding water. Histopathologic analysis revealed the presence of these bacteria in the tissues apparently causing septicaemia and death (Ooi et al., 2020). Overall, the studies to date suggest that only in some cases do changes in bacterial number and populations in haemolymph and other tissues result from their invasion across the damaged cuticle but greater clarification of both the nature and activities of these bacteria is required. Additionally, it is evident that the haemolymph of many apparently healthy aquatic invertebrates is not usually sterile and the presence of small numbers of bacteria in this fluid is not an indicator of disease (e.g., Dupont et al., 2020). Indeed, some of these bacteria may be acting as ‘probiotic’ commensals interfering with the growth of pathogenic forms (Desriac et al., 2014; Quinn, Somolowitz, & Chistoserdov, 2013; Zhang et al., 2018).

Shell disease can cause host death by means other than septicaemia. For example, individuals with extensive lesions can become compromised during the moult when the old, damaged cuticle does not come away from the newly formed cuticle (i.e., dysecdysis; Vogan et al., 1999). These animals are hence unable to fully shed their old cuticle leaving them subject to predation and death.

## CONCLUDING REMARKS

Our understanding of shell disease aetiology has changed dramatically over the last couple of decades from one based on the assumption that this condition is caused by a single, but unknown, agent to our current viewpoint where most forms of shell disease result from dysbiosis of the normal (healthy?) bacterial flora on the outer layer of the cuticle. *Aquimarina* spp. and other *Flavobacteriaceae* are associated with several forms of shell disease in different crustaceans but whether they are primary colonizers is unknown. How environmental changes, such as temperature, can transform microbial community structure to reduced bacterial diversity, and perhaps a shift from a community of commensal bacteria to those with invasive ability, is still unanswered. We suggest that a focus on the virulence factors of isolates including *Aquimarina*, such as adhesin‐like molecules, extracellular enzyme production including chitinases and proteinases, may bring us closer to an explanation of the initiation events in shell disease in situ, and against this background, we caution the reader to scrutinize the limitations of laboratory‐based reports of shell disease progression. Finally, why this condition affects some crustaceans but not others, remains unresolved.

## AUTHOR CONTRIBUTIONS

**Andrew Frederick Rowley:** Conceptualization (equal); funding acquisition (equal); writing – original draft (equal); writing – review and editing (equal). **Christopher James Coates:** Conceptualization (equal); methodology (equal); writing – original draft (equal); writing – review and editing (equal).

## CONFLICT OF INTEREST STATEMENT

The authors declare that they have no conflict of interest.
